# Incidence of Newborn Drug Testing and Variations by Birthing Parent Race and Ethnicity Before and After Recreational Cannabis Legalization

**DOI:** 10.1001/jamanetworkopen.2023.2058

**Published:** 2023-03-08

**Authors:** Sebastian Schoneich, Melissa Plegue, Victoria Waidley, Katharine McCabe, Justine Wu, P. Paul Chandanabhumma, Carol Shetty, Christopher J. Frank, Lauren Oshman

**Affiliations:** 1 Department of Family Medicine, University of Michigan, Ann Arbor; 2 Department of Pediatrics, Michigan Medicine, University of Michigan, Ann Arbor; 3 Department of Family Medicine, University of California, San Diego; 4 Reilly Center for Science, Technology, and Values, University of Notre Dame, South Bend, Indiana; 5 Institute for Healthcare Policy and Innovation, University of Michigan, Ann Arbor

## Abstract

**Importance:**

Thirty-seven US states and the District of Columbia mandate reporting newborns with suspected prenatal substance exposure to the state, and punitive policies that link prenatal substance exposure to newborn drug testing (NDT) may lead to disproportionate reporting of Black parents to Child Protective Services. The impact of recreational cannabis legalization on racial disproportionality in NDT is unknown.

**Objectives:**

To examine variations in the incidence and results of NDT by birthing parent race and ethnicity, variables associated with variation, and changes after statewide legalization of recreational cannabis.

**Design, Setting, and Participants:**

This retrospective cohort study was conducted from 2014 to 2020 with 26 366 live births to 21 648 birthing people who received prenatal care at an academic medical center in the Midwestern United States. Data were analyzed from June 2021 to August 2022.

**Exposures:**

Variables included birthing parent age, race, ethnicity, marital status, zip code, insurance type, prenatal and newborn diagnoses codes, and prenatal urine drug test orders and results.

**Main Outcome and Measures:**

The primary outcome was an NDT order. Secondary outcomes were substances detected.

**Results:**

Among 26 366 newborns of 21 648 birthing people (mean [SD] age at delivery, 30.5 [5.2] years), most birthing parents were White (15 338 [71.6%]), were non-Hispanic (20 125 [93.1%]), and had private insurance coverage (16 159 [74.8%]). The incidence of NDT ordering was 4.7% overall (1237 newborns). Clinicians ordered more NDTs for Black compared with White newborns (207 of 2870 [7.3%] vs 335 of 17 564 [1.9%]; *P* < .001) when the birthing parent had no prenatal urine drug test, a presumably low-risk group. Overall, 471 of 1090 NDTs (43.3%) were positive for only tetrahydrocannabinol (THC). NDTs were more likely to be positive for opioids in White compared with Black newborns (153 of 693 [22.2%] vs 29 of 308 [9.4%]; *P* < .001) and more likely to be positive for THC in Black compared with White newborns (207 of 308 [67.2%] vs 359 of 693 [51.8%]; *P* < .001). Differences remained consistent after state recreational cannabis legalization in 2018. Newborn drug tests were more likely to be positive for THC after legalization vs before legalization (248 of 360 [68.9%] vs 366 of 728 [50.3%]; *P* < .001) with no significant interaction with race and ethnicity groups.

**Conclusions and Relevance:**

In this study, clinicians ordered NDTs more frequently for Black newborns when no drug testing was done during pregnancy. These findings call for further exploration of how structural and institutional racism contribute to disproportionate testing and subsequent Child Protective Services investigation, surveillance, and criminalization of Black parents.

## Key Points

**Question:**

Do newborn drug testing rates and results differ by birthing parent race and ethnicity at a Midwestern US academic medical center, and did this change after state recreational cannabis legalization?

**Findings:**

In a cohort study of 26 366 births from 2014 to 2020, clinicians were more likely to order drug tests for Black newborns (7.3%) compared with White newborns (1.9%) and other racial and ethnic groups when there was no obstetric urine drug test performed during the pregnancy. There was no difference in testing rates or racial inequity after legalization in 2018, but test results were more likely to be positive for tetrahydrocannabinol.

**Meaning:**

This study identified racial inequities in newborn drug testing and calls for an exploration of structural and institutional racism as contributing factors to differences in testing patterns.

## Teaser

This cohort study examines variations in the incidence and results of newborn drug testing by birthing parent race and ethnicity before and after legalization of recreational cannabis in Michigan.

## Introduction

The US Child Abuse and Prevention Treatment Act (CAPTA) requires that all states have policies to identify newborns exposed to substances.^1^ Thirty-seven states and the District of Columbia require clinicians to report suspected prenatal drug use to the state.^2^ The American College of Obstetricians and Gynecologists opposes criminalization of substance use during pregnancy and the use of biologic testing of newborns as a proxy for child abuse or neglect.^3^ Clinicians were found to report Black parents to Child Protective Services (CPS) for prenatal substance use at higher rates than their White counterparts.^4^ This difference has been found in both selective and universal screening programs despite similar prevalence of drug use between the 2 groups.^5,6,7^ While CPS reports can facilitate referral to substance treatment programs and social services, they can also lead to emotional, social, and legal repercussions, including termination of parental rights.^7,8,9^

Prior cross-sectional studies found that clinicians are more likely to ask Black people about substance use in pregnancy and test Black newborns for substance exposure.^10,11,12,13^ A study of urine toxicology testing for hospitalized pregnant people demonstrated more frequent testing for Black and Hispanic people for reasons other than history of substance use.^14^ Studies examining newborn testing have focused on newborns in neonatal intensive care units receiving care for prematurity, those with symptoms suggestive of substance withdrawal, or both, and only one was performed in a state after recreational legalization of cannabis.^11,12,13^ Increasing rates of cannabis use may expose more parents to criminalization of a behavior that is now legal in many states and exacerbate existing racial disparities, but there are no studies investigating racial bias in newborn drug testing before and after recreational cannabis legalization.^15,16,17^

This study aimed to determine the incidence and results of newborn drug testing and variations by birthing parent race and ethnicity at an academic medical center. Secondary aims were to examine incidence of drug tests positive for tetrahydrocannabinol (THC) before and after statewide recreational cannabis legalization and to explore factors associated with variations in order patterns by race and ethnicity. We hypothesized that clinicians would be more likely to test Black newborns compared with non-Hispanic White newborns, that most positive newborn drug tests would be positive only for THC, and that testing would decrease after cannabis legalization.

## Methods

### Study Setting

This retrospective cohort study was performed at a tertiary academic medical center in a Midwestern US state that legalized recreational cannabis on December 6, 2018. Throughout the study period, the institution had no formal policy regarding prenatal urine drug testing or newborn drug testing, leaving testing decisions to the discretion of clinicians. The state mandates that clinicians place a CPS report of suspected child abuse if a newborn has known or suspected exposure to alcohol and/or a controlled substance as evidenced by a positive newborn drug test or withdrawal symptoms.^18^ The University of Michigan institutional review board approved this study and waived the requirement for patient consent, per the Common Rule. This study followed the Strengthening the Reporting of Observational Studies in Epidemiology (STROBE) reporting guideline for cohort studies.^19^

### Study Sample

Pregnancies resulting in a live birth at the study institution were eligible for inclusion. We obtained electronic health record (EHR) data on 27 647 live births from July 1, 2014, to December 31, 2020. We excluded birth records from 1281 birthing people who did not receive prenatal care at the study institution due to lack of available data on relevant analytical variables.

### Study Approach

Our study uses a dyadic approach, in which maternal and infant health disparities are intertwined and systematic racism is understood to be a shared causal pathway for Black maternal morbidity and mortality and disproportionate referrals of Black families to CPS for prenatal substance exposure.^20^ We build on the work of antiracist scholars and scholars who belong to minoritized racial and ethnic groups and are guided by an antiracist and justice-informed praxis.^21,22,23^

### Study Measures

The primary outcome variable was a newborn drug test order. The unit of analysis was a single live birth. For births with multiples, a test order for any newborn in the same delivery was considered an order for the birth. A secondary outcome was substance(s) detected, if any, in the newborn drug test. A meconium enzyme-linked immunosorbent assay test that screens for amphetamines, cannabinoids, cocaine and metabolites, opiates, and phencyclidine was used; a positive screen automatically reflexes to confirmatory liquid chromatography–tandem mass spectrometry testing.

Demographic data included birthing parent age, self-reported race, ethnicity, marital status, insurance type, and zip code. The EHR did not capture self-reported gender; accordingly we use the gender inclusive term birthing parent.^24^ Additional diagnosis variables abstracted for each newborn and billing and problem list diagnosis codes for the birthing parent from delivery and prenatal encounters included *International Classification of Diseases, Ninth Revision *(*ICD-9*) and *International Statistical Classification of Diseases and Related Health Problems, Tenth Revision *(*ICD-10*) diagnostic codes for alcohol, tobacco, opioid-related disorders, other substance use, and placental abruption (eTable 1 in Supplement 1).

Test orders and results for newborn and prenatal drug tests were collected and reported by the substances and drug classes tested in each panel. Newborn results were recorded as positive or negative for amphetamine, cocaine, methamphetamine, opioids, phencyclidine, and THC. Prenatal urine drug test results were recorded in a similar fashion for amphetamines, barbiturates, benzodiazepines, buprenorphine, cocaine, cannabinoids, methadone, methamphetamine, opiates, and oxycodone. Prenatal results were aggregated into 3 categories for statistical analysis: patients who were not tested; those tested at least once with a negative result for amphetamines, buprenorphine, cannabinoids, cocaine, methamphetamines, methadone, opiates, and oxycodone; and those tested at least once with positive results for 1 or more of the previously listed controlled substances. Patients with active prescriptions for buprenorphine or methadone who had tests positive only for their prescribed medication were reported with the negative result group. We excluded barbiturates and benzodiazepines, which were typically medically prescribed, in the included sample of patients.

Categories of patient self-reported race were American Indian or Alaska Native, Asian, Black, Native Hawaiian or other Pacific Islander, other, and White. Patient self-reported ethnicity was listed as Hispanic or non-Hispanic. When referring to newborn drug testing, we reported newborn race concordant with birthing parent self-reported race. We categorized patients self-reporting more than 1 race as multiracial. In all analyses, we aggregated data for American Indian or Alaska Native (48 birthing parents) and Native Hawaiian or other Pacific Islander (22 birthing parents) with patients who self-selected other race given small sample sizes and refer to this as a combined group. In regression analyses, we further merged patients who self-reported as Asian with American Indian or Alaska Native, Native Hawaiian or other Pacific Islander and other race into this combined group, as they had similarly low newborn drug testing rates.

We mapped the birthing parent zip code to 2010 US Census tract data to collect the neighborhood disadvantage index (NDI) using data from the Neighborhood Data Archive, which ranges from 0 to 100 with a higher number representing greater disadvantage.^25^ The NDI includes the proportion of households in the census tract that are female-headed with children, receiving public assistance or food stamps, and have income below the federal poverty level as well as the proportion of unemployed among those aged 16 years or older. This measure excludes individual race and can be used as a proxy measure for exposure to structural racism at the neighborhood level.^23^ We categorized NDI into less than 10, 10 to 19, and 20 or greater for statistical analysis to compare the highest to lowest advantage neighborhoods within our sample.

### Statistical Analysis

Statistical analyses were performed using Stata version 17.0 (StataCorp) and R version 4.1.2 (R Project for Statistical Computing) with a specified 2-tailed α level of .05. We report birthing parent demographic variables using descriptive statistics stratified by newborn drug test order. The rate of newborn drug test orders was stratified by race and ethnicity, by year during the study period (2014-2020), before and after recreational cannabis legalization, and by prenatal urine drug test order and result. We used logistic regression models to compare newborn drug test incidence estimates including race or ethnicity, the stratifying variable (time or urine drug test), and the interaction as covariates.

We performed univariate analysis using logistic regression for newborn drug test order with demographic variables and diagnostic variables related to tobacco, alcohol, opioid, substance use, placental abruption, and prenatal urine drug test results as single covariates. Adjusted associations were evaluated by including all covariates in the same model. Interactions between race or ethnicity and other covariates were assessed and retained if significant. We performed secondary analyses to assess how cannabis legalization affected associations by including a dichotomous indicator for births occurring before and after legalization date interacting with key variables.

We analyzed newborn drug test results by comparing the proportion of positive tests for each substance class across race and ethnicity groups. We then used a logistic regression model with THC result as the outcome and a dichotomous indicator for births occurring before or after legalization as a covariate.

All models were run under a generalized estimating equation (GEE) framework to account for correlations present between multiple deliveries for the same birthing parent during the study period. Models assumed an exchangeable correlation matrix and estimated robust standard errors.

## Results

There were 27 647 live births during the study period, of which 1281 (4.6%) were excluded from analysis due to a lack of prenatal care within the medical center. Of the remaining 26 366 births, 25 701 (97.5%) were singleton and 13 646 (51.8%) were male sex. Most of the 21 648 birthing parents (mean [SD] age at delivery, 30.5 [5.2] years) were White (15 338 [71.6%]), were non-Hispanic (20 125 [93.1%]), and had private insurance coverage (16 159 [74.8%]). A newborn drug test order was placed for 1237 newborns (4.7%), including 358 of 3164 Black newborns (11.3%), 775 of 18 372 White newborns (4.2%), 44 of 1267 Hispanic newborns (3.5%), 22 of 338 multiracial newborns (6.5%), 4 of 2379 Asian newborns (0.2%), and 21 of 567 newborns (3.7%) of American Indian and Alaska Native, Native Hawaiian and other Pacific Islander, and other self-reported race (combined group) (Table 1).

**Table 1.** Birthing Parent Demographic Characteristics by Newborn Drug Test Order Status

Characteristic	Newborn drug test, No. (%)	
	No order (n = 25 129)	Order (n = 1237)
Age at delivery, mean (SD), y	30.63 (5.2)	27.22 (5.5)
Race		
American Indian or Alaska Native	43 (0.2)	5 (0.4)
Asian	2384 (9.5)	4 (0.3)
Black	2845 (11.3)	359 (29.0)
Multiracial	364 (1.4)	31 (2.5)
Native Hawaiian or other Pacific Islander	22 (0.1)	0
Other^a^	1107 (4.4)	28 (2.3)
White	18 088 (72.0)	797 (64.4)
Missing	276 (1.1)	13(1.1)
Ethnicity		
Not Hispanic	23 372 (93.0)	1175 (95.0)
Hispanic	1223 (4.9)	44 (3.6)
Missing	534 (2.1)	18 (1.5)
Marital status		
Single	5992 (23.8)	777 (62.8)
Married or single other	15 413 (61.3)	214 (17.3)
Divorced or separated	248 (1.0)	30 (2.4)
Other or widowed	23 (0.1)	2 (0.2)
Missing	3453 (13.7)	214(17.3)
Insurance		
Private	19 139 (76.2)	582 (47.0)
Medicaid	3684 (14.7)	501 (40.5)
Medicare	111 (0.4)	43 (3.5)
Other public program	733 (2.9)	15 (1.2)
Self pay	1462 (5.8)	96 (7.8)
Neighborhood Disadvantage Index^b^		
<10	16 606 (66.1)	488 (39.5)
10 to 19	4991 (19.9)	415 (33.5)
≥20	1515 (6.0)	252 (20.4)
Missing	2017 (8.0)	82 (6.6)

^a^ Individuals who self-selected other as a race identifier in the electronic health record.

^b^ Neighborhood disadvantage index was calculated using data from the Neighborhood Data Archive, which ranges from 0 to 100 with a higher number representing greater disadvantage. The neighborhood disadvantage index was categorized into less than 10, 10 to 19, and 20 or greater for statistical analysis.

**Figure 1.**
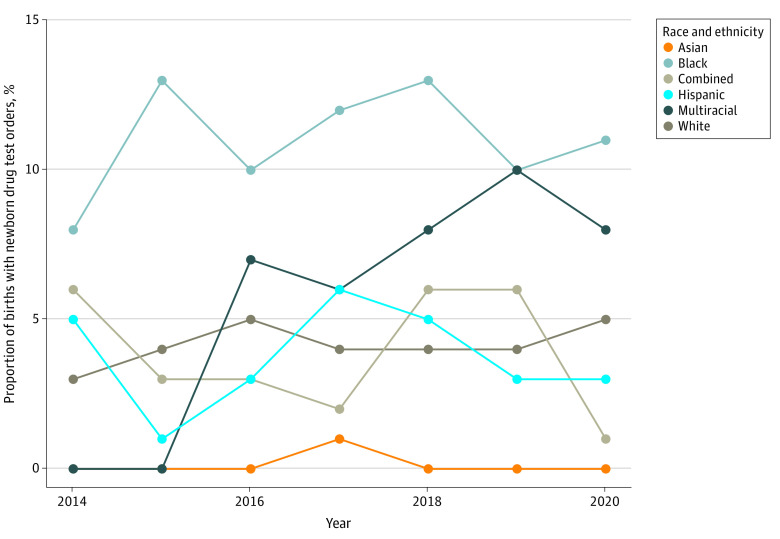
Newborn Drug Testing Incidence Over Time, by Birthing Parent Race and Ethnicity Newborn drug testing incidence was significantly higher for Black newborns compared with White and Asian newborns in all years, newborns in the combined group (those self-reporting as American Indian or Alaska Native, Native Hawaiian or other Pacific Islander, and other race) for all years except 2014 and 2019, and Hispanic newborns in all years except 2014. Testing prevalence was significantly higher for White newborns compared with Asian newborns in all years, the combined group in 2020, multiracial newborns in 2014 and 2015, and Hispanic newborns in 2015. Multiracial includes patients self-reporting as 2 or more race options. Hispanic includes patients self-reporting as Hispanic ethnicity, regardless of race selection.

Newborn drug tests were ordered significantly more frequently for Black newborns except for 2016 to 2020, when they did not differ from the multiracial group only (Figure 1). There was no significant difference in the rate of newborn drug tests ordered before and after state recreational cannabis legalization on December 6, 2018, overall or within race and ethnicity groups. In 2016, clinicians ordered tests for 49 of 470 Black newborns (10.4%) compared with 132 of 2695 White (4.9%; *P* = .001) and 4 of 57 multiracial (7.0%; *P* = .29) newborns. In comparison, in 2019 clinicians ordered tests for 115 of 3001 White newborns (3.8%) compared with 52 of 507 Black newborns (10.3%; *P* < .001) and 7 of 69 multiracial newborns (10.1%; *P* = .90 vs Black newborns).

In an unadjusted GEE model, Black newborns had 172% higher odds of a newborn drug test order compared with White newborns (odds ratio, 2.72; 95% CI, 2.37-3.13). Black newborns had higher odds of a newborn drug test order than all other race and ethnicity groups (Table 2 and eTable 2 in Supplement 1).

**Table 2.** Results of Unadjusted and Adjusted Generalized Estimating Equation Logistic Regression Models for Newborn Drug Test Orders

Characteristic	Univariate model		Adjusted model	
	OR (95% CI)	*P* value	OR (95% CI)	*P* value
Birthing parent				
Age	0.89 (0.88-0.90)	<.001	0.94 (0.92-0.96)	<.001
Race and ethnicity				
White	1 [Reference]	NA	1 [Reference]	NA
Black	2.72 (2.37-3.13)	<.001	1.31 (1.04-1.66)	.02
Combined^a^	0.17 (0.11-0.27)	<.001	0.64 (0.38-1.07)	.09
Hispanic	0.78 (0.56-1.07)	.13	0.89 (0.56-1.42)	.62
Multiracial^b^	1.58 (1.00-2.48)	.049	0.79 (0.38-1.62)	.52
Marital status				
Married	1 [Reference]	NA	1 [Reference]	NA
Single	9.12 (7.75-10.71)	<.001	2.89 (2.31-3.62)	<.001
Other	8.19 (5.48-12.24)	<.001	2.81 (1.51-5.26)	.001
Insurance				
Private	1 [Reference]	NA	1 [Reference]	NA
Public	3.69 (3.26-4.17)	<.001	1.44 (1.19-1.75)	<.001
Self pay	2.02 (1.63-2.51)	<.001	1.33 (0.93-1.90)	.14
Tobacco diagnosis				
No	1 [Reference]	NA	1 [Reference]	NA
Yes	13.42 (11.80-15.26)	<.001	3.15 (2.56-3.87)	<.001
Alcohol diagnosis				
No	1 [Reference]	NA	1 [Reference]	NA
Yes	12.41 (9.20-16.73)	<.001	1.55 (0.92-2.63)	.10
Other substance use diagnosis				
No	1 [Reference]	NA	1 [Reference]	NA
Yes	83.63 (72.03-97.10)	<.001	15.77 (12.76-19.49)	<.001
Placental abruption diagnosis				
No	1 [Reference]	NA	1 [Reference]	NA
Yes	1.96 (1.41-2.71)	<.001	1.38 (0.77-2.46)	.28
Prenatal urine drug test				
None	1 [Reference]	NA	1 [Reference]	NA
Illicit substance^c^				
Negative	17.54 (14.51-21.21)	<.001	3.49 (2.63-4.63)	<.001
Positive	101.92 (83.64-124.20)	<.001	12.36 (9.20-16.59)	<.001
Neighborhood Disadvantage Index^d^				
≤10	1 [Reference]	NA	1 [Reference]	NA
10-20	2.71 (2.35-3.12)	<.001	1.58 (1.27-1.96)	<.001
20+	5.53 (4.67-6.54)	<.001	1.83 (1.37-2.42)	<.001

Abbreviations: NA, not applicable; OR, odds ratio.

^a^ Combined group includes patients self-reporting as American Indian or Alaska Native, Asian, Native Hawaiian or other Pacific Islander, and other race.

^b^ Multiracial includes patients self-reporting as 2 or more race options.

^c^ lllicit substances include amphetamines, buprenorphine, cannabinoids, cocaine, methamphetamines, methadone, opiates, and oxycodone.

^d^ Neighborhood disadvantage score was calculated using data from the Neighborhood Data Archive, which ranges from 0 to 100 with a higher number representing greater disadvantage. The neighborhood disadvantage index was categorized into less than 10, 10 to 19, and 20 or greater for statistical analysis.

Urine tests were ordered for 1206 of 26 366 pregnancies (4.6%); 294 (9.1%) for Black, 808 (4.5%) for White, 27 (8.0%) for multiracial, 42 (3.3%) for Hispanic, and 25 (0.8%) for pregnancies in the combined group (*P* < .001). The newborn drug test order rate was 2.4% when no prenatal urine drug test was performed (594 of 25 160 pregnancies), 32.2% for births with a negative urine drug test (204 of 634 pregnancies), and 76.8% when the urine drug test was positive (439 of 572 pregnancies; *P* < .001). A GEE model including interaction between race and ethnicity and prenatal urine drug testing found the association between race and ethnicity and newborn drug test order rates varied across prenatal urine drug test results (*P *for interaction < .001).

**Figure 2.**
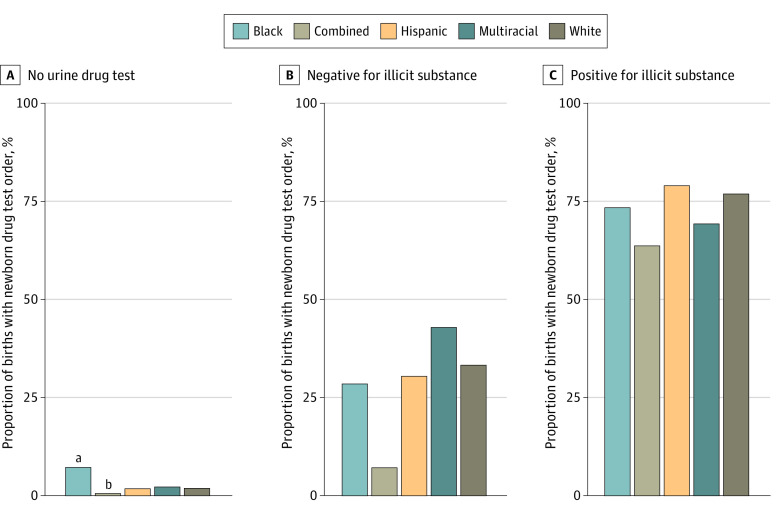
Newborn Drug Testing Rates by Birthing Parent Race and Ethnicity and by Prenatal Urine Drug Test Order and Result The combined group includes patients self-reporting as American Indian or Alaska Native, Native Hawaiian or other Pacific Islander, Asian, and other race. Illicit substances include amphetamines, buprenorphine without prescription, cannabinoids, cocaine, methamphetamines, methadone without prescription, opiates, and oxycodone, which were typically nonprescribed controlled substances. Barbiturates and benzodiazepines were not included in this analysis because these were typically prescribed (legal) controlled substances. ^a^Significantly higher than White (*P* < .001), combined (*P* < .001), multiracial (*P* < .001), and Hispanic (*P* < .001) newborns. ^b^Significantly lower than Black (*P* < .001) and White (*P* < .001) newborns.

Clinicians were more likely to test Black newborns (207 of 2870 [7.3%]) compared with multiracial (7 of 311 [2.3%]), White (335 of 17 564 [1.9%]), Hispanic (22 of 1225 [1.8%]), or the combined group (17 of 2921 [0.6%]; *P* < .001) when there was no urine drug test performed during the pregnancy. However, there was no difference by race or ethnicity when the prenatal urine drug test was performed, whether the results were positive or negative (Figure 2).

In an adjusted GEE model, Black newborns had 31% higher odds of a newborn drug test order compared with White newborns (adjusted odds ratio, 1.31; 95% CI, 1.04-1.66) (Table 2). In other words, White newborns were 24% less likely to have a newborn drug test ordered compared with Black newborns. There were no significant interactions found between race and ethnicity groups and other factors in the model. Secondary analyses including interactions with pre– and post–cannabis legalization time period ddid not change associations found with race and ethnicity.

Among 1090 resulted newborn drug tests, THC was found in 614 (56.3%), opioids in 196 (18.0%), and in less than 6% each for amphetamine, cocaine, methamphetamine, and phencyclidine. Overall, 30.0% of tests were found to be negative for all substances (328), 54.0% positive for a single substance (588), and 15.8% positive for 2 or more (172); these findings did not differ across race and ethnicity groups (*P* = .32). A total of 471 newborn drug tests (43.2%) were positive for THC only. Newborn drug tests were more likely to be positive for THC after legalization vs before legalization (248 of 360 [68.9%] vs 366 of 728 [50.3%]; *P* < .001) with no significant interaction with race and ethnicity groups.

**Figure 3.**
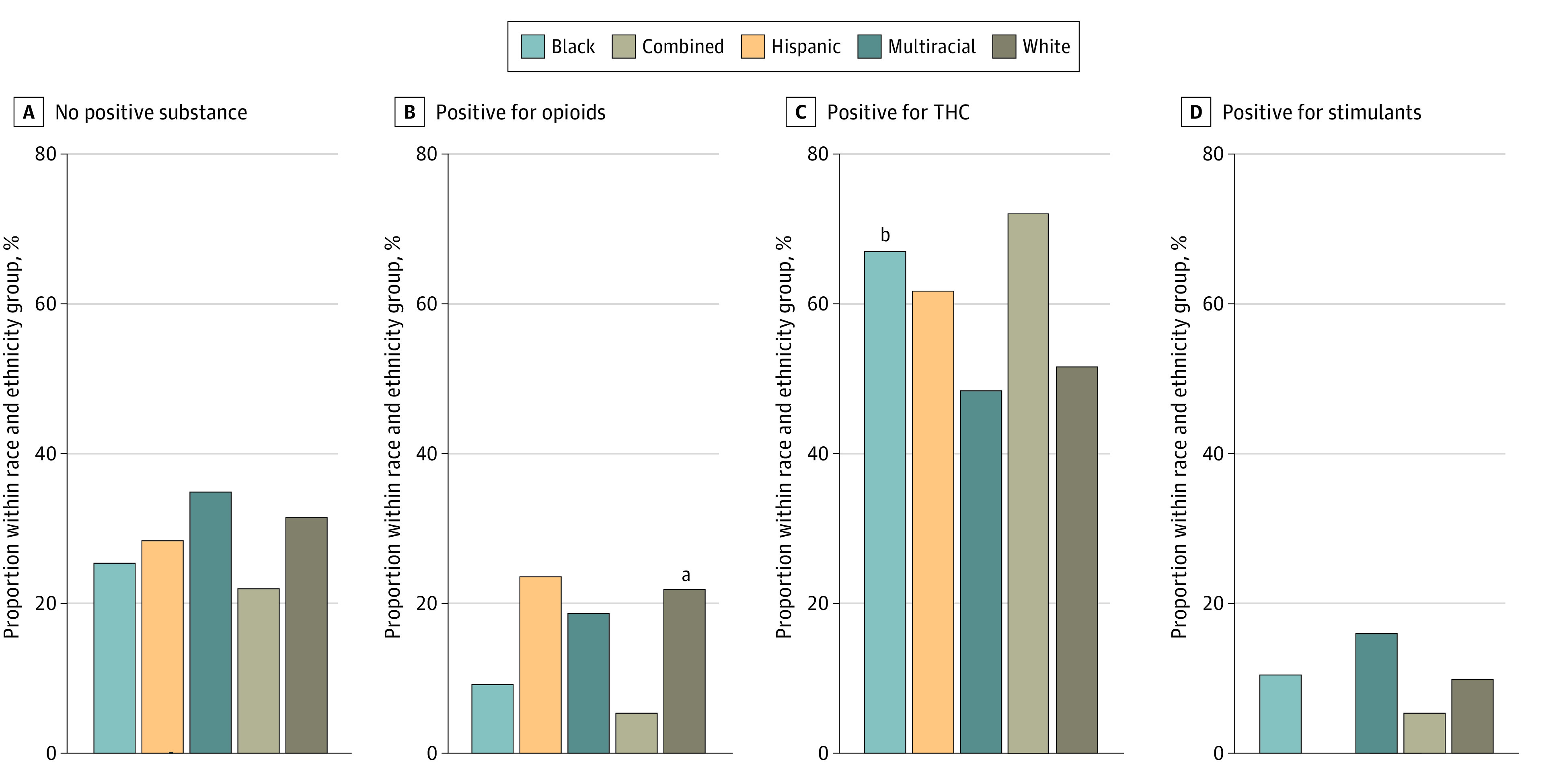
Newborn Drug Test Results by Substance and Birthing Parent Race and Ethnicity The combined group includes patients self-reporting as American Indian or Alaska Native, Asian, Native Hawaiian or other Pacific Islander, and other race. Multiracial includes patients self-reporting as 2 or more race options. Opioids include morphine, oxymorphone, codeine, oxycodone, and hydrocodone. Stimulants include amphetamines, cocaine, and methamphetamine. ^a^Significantly higher than Black newborns (*P* < .001). ^b^Significantly higher than White newborns (*P* < .001).

Patterns of positive substances were assessed among race and ethnicity groups (Figure 3). Tests were significantly more likely to be positive for opioids among White compared with Black newborns (153 of 693 [22.2%] vs 29 of 308 [9.4%]; *P* < .001*)* and significantly more likely to be positive for THC among Black compared with White newborns (207 of 308 [67.2%] vs 359 of 693 [51.8%]; *P* < .001). Differences remained consistent when assessed before and after recreational cannabis legalization.

## Discussion

This retrospective cohort study examined the incidence of newborn drug testing and variations by birthing parent race and ethnicity at a single academic medical center in the Midwestern US before and after statewide recreational cannabis legalization. The unadjusted and adjusted odds of a clinician ordering a drug test for Black newborns were greater than for newborns of all other race and ethnicity groups, except multiracial newborns, but this difference only persisted when urine drug testing was not performed during prenatal care, a presumably low-risk group. The racial disparity in newborn drug testing did not change after legalization of recreational cannabis, but test results overall were more likely to be positive for THC.

Four prior studies performed at hospitals in the Northeast United States with data spanning 1998 to 2018 found higher drug testing rates for Black newborns. All had smaller cohorts, 1 limited to patients with Medicaid insurance, 2 limited to newborns admitted to the neonatal intensive care unit, and 1 with limited access to prenatal records.^10,11,12,13^ These studies raised the question of whether higher rates of drug testing for Black newborns may be a racial disparity related to implicit bias on the part of clinicians. A major strength of our study is the large cohort size and 7 years of complete, linked prenatal and newborn data spanning recreational cannabis legalization in our state. Our study adds to evidence suggesting that structural and institutional racism contribute to overtesting Black newborns and possible subsequent overreporting to CPS.

Cannabis use in pregnancy has been increasing, with up to 12.1% of people reporting use in the first trimester in 2016 to 2017.^17^ In our study population, 43.2% of newborn drug tests were positive only for THC, Black newborns were more likely to have a drug test positive for THC than White newborns, and tests positive for THC increased after legalization across all race and ethnicity groups. These findings suggest higher overall cannabis use among pregnant people in our study sample after legalization. Despite a lack of studies demonstrating that cannabis use in pregnancy increases the risk of child abuse or neglect, recreational cannabis remains a federally controlled substance, and state law where this study was conducted mandates a CPS report for a newborn drug test positive for THC.^26^

Prior studies show that pregnant patients who belong to minoritized racial and ethnic groups are more likely to receive urine drug testing during pregnancy when they verbally report substance use.^14^ Our institution did not require universal prenatal urine testing to confirm verbal reports. We report low rates of urine testing (4.6%) with higher rates of testing for Black and multiracial pregnant people. Our finding that clinician rates of newborn drug testing were higher in Black newborns only when no urine drug testing was done suggests that newborn clinicians may be inequitably applying state CAPTA laws to Black birthing people, leading to inequitable rates of CPS referrals and downstream harms of surveillance and criminalization of parental drug use.^5,27,28^ The concept of obstetrical racism—defined as forms of violence that medical personnel perpetrate against Black birthing people—situates overtesting of Black newborns within the larger epidemic of Black maternal morbidity and mortality and the criminalization and policing of Black parents.^15,29,30^

In the adjusted regression analysis, White newborns were 24% less likely to receive a newborn drug test compared with Black newborns but were more likely to have a drug test positive for opioids. This suggests that clinician implicit biases may lead to undertesting of White newborns and a missed opportunity to detect and treat opioid use disorder.^16^ We view overtesting of Black newborns as a legacy of the culture of White supremacy in reproductive health care in which the conduct of Black women, specifically regarding prenatal substance exposure, is criminalized via the CPS system.^31,32^ Our findings strongly suggest that changes in policies regarding drug testing and reporting at the hospital and state level and improvement measures focused on the health, well-being, and dignity of Black birthing people are needed to reduce health inequity for Black parents and their newborns.^33,34^

### Limitations

This study has limitations. Our findings from an academic medical center in the Midwestern US may not be generalizable to other patient populations. Our sample had lower rates of public insurance and excluded patients with no prenatal care or prenatal care outside the medical center; the excluded populations may have had differing rates of substance use and newborn drug testing. We did not capture medical cannabis prescription data, amphetamine prescriptions, ordering clinician characteristics, or indications for testing, and we did not assess scant prenatal care, comorbid pain, and psychiatric conditions, which may reflect unmeasured decision-making factors. The study institution does not require universal prenatal urine testing and not all prenatal urine drug tests were subjected to reflex confirmatory testing, so the true prevalence of substance use in pregnancy may be higher than estimated in this study.^35^

## Conclusions

This study expands on prior studies that have found racial disparities in newborn drug testing unrelated to obstetrical risk. Future approaches that dissociate the medical treatment of substance use disorders from mandated state reporting and those that center justice for Black mothers and babies and their families are urgently needed to break the cycle of scrutiny, criminalization, and separation of Black families.^15,33^

## Notes

Supplement 1. **eTable 1. **Diagnosis Codes **eTable 2.** Alternative Race and Ethnicity Group Comparisons for GEE Logistic Regression Models on Newborn Drug Test Orders

Supplement 2.
Data Sharing Statement

## References

[1] Children’s Bureau. About CAPTA: a legislative history. February 2019. Accessed January 31, 2023. https://www.childwelfare.gov/pubpdfs/about.pdf

[2] McCOURT AD, White SA, Bandara S, . Development and implementation of state and federal child welfare laws related to drug use in pregnancy. Milbank Q. 2022;100(4):1076-1120. doi:10.1111/1468-0009.12591 36510665 PMC9836249

[3] American College of Obstetricians and Gynecologists. Opposition to criminalization of individuals during pregnancy and the postpartum period. Accessed February 26, 2021. https://www.acog.org/clinical-information/policy-and-position-statements/statements-of-policy/2020/opposition-criminalization-of-individuals-pregnancy-and-postpartum-period

[4] Chasnoff IJ, Landress HJ, Barrett ME. The prevalence of illicit-drug or alcohol use during pregnancy and discrepancies in mandatory reporting in Pinellas County, Florida. N Engl J Med. 1990;322(17):1202-1206. doi:10.1056/NEJM199004263221706 2325711

[5] Terplan M, Minkoff H. Neonatal abstinence syndrome and ethical approaches to the identification of pregnant women who use drugs. Obstet Gynecol. 2017;129(1):164-167. doi:10.1097/AOG.0000000000001781 27926654

[6] Roberts SCM, Nuru-Jeter A. Universal alcohol/drug screening in prenatal care: a strategy for reducing racial disparities? questioning the assumptions. Matern Child Health J. 2011;15(8):1127-1134. doi:10.1007/s10995-010-0720-6 21107668 PMC3135764

[7] Roberts SCM, Zahnd E, Sufrin C, Armstrong MA. Does adopting a prenatal substance use protocol reduce racial disparities in CPS reporting related to maternal drug use? a California case study. J Perinatol. 2015;35(2):146-150. doi:10.1038/jp.2014.168 25233193

[8] Cooper NM, Lyndon A, McLemore MR, Asiodu IV. Social construction of target populations: a theoretical framework for understanding policy approaches to perinatal illicit substance screening. Policy Polit Nurs Pract. 2022;23(1):56-66. doi:10.1177/15271544211067781 34939864 PMC9017642

[9] Austin AE, Naumann RB, Simmons E. Association of state child abuse policies and mandated reporting policies with prenatal and postpartum care among women who engaged in substance use during pregnancy. JAMA Pediatr. 2022;176(11):1123-1130. doi:10.1001/jamapediatrics.2022.3396 36121649 PMC9486638

[10] Kerker BD, Leventhal JM, Schlesinger M, Horwitz SM. Racial and ethnic disparities in medical history taking: detecting substance use among low-income pregnant women. Ethn Dis. 2006;16(1):28-34. 16599345

[11] Ellsworth MA, Stevens TP, D’Angio CT. Infant race affects application of clinical guidelines when screening for drugs of abuse in newborns. Pediatrics. 2010;125(6):e1379-e1385. doi:10.1542/peds.2008-3525 20478941

[12] Kunins HV, Bellin E, Chazotte C, Du E, Arnsten JH. The effect of race on provider decisions to test for illicit drug use in the peripartum setting. J Womens Health (Larchmt). 2007;16(2):245-255. doi:10.1089/jwh.2006.0070 17388741 PMC2859171

[13] Perlman NC, Cantonwine DE, Smith NA. Toxicology testing in a newborn ICU: does social profiling play a role? Hosp Pediatr. 2021;11(9):e179-e183. doi:10.1542/hpeds.2020-005765 34373267

[14] Perlman NC, Cantonwine DE, Smith NA. Racial differences in indications for obstetrical toxicology testing and relationship of indications to test results. Am J Obstet Gynecol MFM. 2022;4(1):100453. doi:10.1016/j.ajogmf.2021.100453 34352428

[15] Davis DA. Obstetric racism: the racial politics of pregnancy, labor, and birthing. Med Anthropol. 2019;38(7):560-573. doi:10.1080/01459740.2018.1549389 30521376

[16] McLemore MR, Asiodu I, Crear-Perry J, . Race, research, and women’s health: best practice guidelines for investigators. Obstet Gynecol. 2019;134(2):422-423. doi:10.1097/AOG.0000000000003393 31348216

[17] Volkow ND, Han B, Compton WM, McCance-Katz EF. Self-reported medical and nonmedical cannabis use among pregnant women in the United States. JAMA. 2019;322(2):167-169. doi:10.1001/jama.2019.7982 31211824 PMC6582258

[18] State of Michigan Department of Health and Human Services. Children’s Protective Services Manual: Section PSM 716-7 Cases Involving Substances. Accessed August 21, 2022. https://dhhs.michigan.gov/OLMWEB/EX/PS/Public/PSM/716-7.pdf#pagemode=bookmarks

[19] von Elm E, Altman DG, Egger M, Pocock SJ, Gøtzsche PC, Vandenbroucke JP; STROBE Initiative. The Strengthening the Reporting of Observational Studies in Epidemiology (STROBE) statement: guidelines for reporting observational studies. Lancet. 2007;370(9596):1453-1457. doi:10.1016/S0140-6736(07)61602-X 18064739

[20] Glazer KB, Zeitlin J, Howell EA. Intertwined disparities: applying the maternal-infant dyad lens to advance perinatal health equity. Semin Perinatol. 2021;45(4):151410. doi:10.1016/j.semperi.2021.151410 33865629 PMC8184592

[21] Ford CL, Airhihenbuwa CO. Critical Race Theory, race equity, and public health: toward antiracism praxis. Am J Public Health. 2010;100(Suppl 1)(suppl 1):S30-S35. doi:10.2105/AJPH.2009.171058 20147679 PMC2837428

[22] Ghidei L, Murray A, Singer J. Race, research, and women’s health: best practice guidelines for investigators. Obstet Gynecol. 2019;133(4):815-818. doi:10.1097/AOG.0000000000003157 30870295

[23] Lett E, Asabor E, Beltrán S, Cannon AM, Arah OA. Conceptualizing, contextualizing, and operationalizing race in quantitative health sciences research. Ann Fam Med. 2022;20(2):157-163. doi:10.1370/afm.2792 35045967 PMC8959750

[24] Rioux C, Weedon S, London-Nadeau K, . Gender-inclusive writing for epidemiological research on pregnancy. J Epidemiol Community Health. 2022;76(9):823-827. doi:10.1136/jech-2022-219172 35764388

[25] Melendez R, Clarke P, Khan A, Gomez-Lopez I, Li M, Chenoweth M. National Neighborhood Data Archive (NaNDA): socioeconomic status and demographic characteristics of census tracts, United States, 2008-2017. Published online December 14, 2020. doi:10.3886/E119451V2

[26] Michigan Comp Laws § 333.7212 (2013). Accessed August 21, 2022. http://legislature.mi.gov/doc.aspx?mcl-333-7212

[27] Wakeman SE, Bryant A, Harrison N. Redefining child protection: addressing the harms of structural racism and punitive approaches for birthing people, dyads, and families affected by substance use. Obstet Gynecol. 2022;140(2):167-173. doi:10.1097/AOG.0000000000004786 35852265

[28] Thomas MMC, Waldfogel J, Williams OF. Inequities in child protective services contact between Black and White children. Child Maltreat. 2023;28(1):42-54. doi:10.1177/10775595211070248 35081781 PMC9325927

[29] Bridges K. Race, pregnancy, and the opioid epidemic: White privilege and the criminalization of opioid use during pregnancy. Harv Law Rev. 2020;133(3):771-851. Accessed January 31, 2023. https://harvardlawreview.org/2020/01/race-pregnancy-and-the-opioid-epidemic-white-privilege-and-the-criminalization-of-opioid-use-during-pregnancy/

[30] Admon LK, Winkelman TNA, Zivin K, Terplan M, Mhyre JM, Dalton VK. Racial and ethnic disparities in the incidence of severe maternal morbidity in the United States, 2012-2015. Obstet Gynecol. 2018;132(5):1158-1166. doi:10.1097/AOG.0000000000002937 30303912

[31] Roberts DE. Killing the Black Body: Race, Reproduction, and the Meaning of Liberty. Vintage Books; 1999.

[32] Logan RG, McLemore MR, Julian Z, Stoll K, Malhotra N, Vedam S; GVtM Steering Council. Coercion and non-consent during birth and newborn care in the United States. Birth. 2022;49(4):749-762. doi:10.1111/birt.12641 35737547

[33] Roberts SCM. Good intentions are not enough: truly supporting pregnant women with substance use disorders requires evaluating the impact of our policies and practices. Womens Reprod Health (Phila). 2020;7(3):181-184. doi:10.1080/23293691.2020.1780398

[34] Crear-Perry J, Green, C, Cruz, K. Respectful maternity care: shifting medical education and practice toward an anti-racist framework. Health Aff Forefront. April 16, 2021. doi:10.1377/forefront.20210413.303812

[35] Wexelblatt SL, Ward LP, Torok K, Tisdale E, Meinzen-Derr JK, Greenberg JM. Universal maternal drug testing in a high-prevalence region of prescription opiate abuse. J Pediatr. 2015;166(3):582-586. doi:10.1016/j.jpeds.2014.10.004 25454935

