# Supplementary material for: Incidence of Newborn Drug Testing and Variations by Birthing Parent Race and Ethnicity Before and After Recreational Cannabis Legalization
Source: JAMA Netw Open. 2023 Mar 8;6(3):e232058. doi: 10.1001/jamanetworkopen.2023.2058 (PMC9996400; doi:10.1001/jamanetworkopen.2023.2058)
Supplement: Supplement 1. — eTable 1. Diagnosis Codes eTable 2. Alternative Race and Ethnicity Group Comparisons for GEE Logistic Regression Models on Newborn Drug Test Orders [file jamanetwopen-e232058-s001.pdf]

Supplemental Online Content

Schoneich S, Plegue M, Waidley V, et al. Incidence of newborn drug testing and variations by birthing parent race and ethnicity before and after recreational cannabis legalization. *JAMA Netw Open.* 2023;6(3):e232058. doi:10.1001/jamanetworkopen.2023.2058

eTable 1. Diagnosis Codes

eTable 2. Alternative Race and Ethnicity Group Comparisons for GEE Logistic Regression Models on Newborn Drug Test Orders

This supplemental material has been provided by the authors to give readers additional information about their work.

© 2023 Schoneich S et al. *JAMA Network Open.*

eTable 1. Diagnosis Codes

Diagnosis Type	ICD-10 Codes	ICD-9 Codes
**Alcohol**	F10.1, F10.2, F10.9, G31.2, G62.1, I42.6, K29.2, K70, O35.4, O35.5, O99.31	303, 303.9, 305.0, 357.5, 425.5, 535.3, 571.0, 571.1, 571.2, 571.3, 571.9, 655.4, 760.7
**Tobacco**	F17.2, O99.33, Z72.0	305.1, 649.0
**Other Substance**	F11.1, F11.2, F11.9, F12.1, F12.2, F12.9, F13.1, F13.2, F13.9, F14.1, F14.2, F14.9, F15.1, F15.2, F15.9, F16.1, F16.2, F16.9,F18.1, F18.2, F18.9, F19.1, F19.2, F19.9, O99.32, T40.0, T40.1, T40.2,T40.3, T40.4, T40.6, T40.5, T40.7, T40.8, T40.9,T40.99, T41.0X2, T43.6	304.0, 304.1, 304.2, 304.3, 304.4, 304.7, 304.8, 304.9, 305.2, 305.3, 305.4, 305.5, 305.6, 305.7, 305.9, 779.5, 965.0, 968.1, 968.2, 969.7, 969.9, 970.81
**Placental Abruption**	O45.0, O45.8, O45.9	641.2

© 2023 Schoneich S et al. *JAMA Network Open.*

eTable 2. Alternative[a] Race and Ethnicity Group Comparisons for GEE Logistic Regression Models on Newborn Drug Test Orders

	Univariate Models		Adjusted Model[d]	
	OR [95% CI]	P value	OR [95% CI]	P value
**Birthing Parent Race (ref=Black)**				
White	0.37 [0.32 – 0.42]	<0.001	0.76 [0.60 – 0.96]	0.022
Combined[b]	0.06 [0.04 - 0.10]	<0.001	0.48 [0.28 – 0.84]	0.009
Multiracial[c]	0.58 [0.37 – 0.92]	0.021	0.60 [0.29 – 1.25]	0.173
Hispanic	0.28 [0.20 – 0.40]	<0.001	0.68 [0.42 – 1.10]	0.114
**Birthing Parent Race (ref=Combined[b])**				
White	5.73 [3.74 – 8.79]	<0.001	1.57 [0.93 – 2.66]	0.089
Black	15.60 [10.07 – 24.13]	<0.001	2.07 [1.20 – 3.57]	0.009
Multiracial[c]	9.04 [4.89 – 16.70]	<0.001	1.24 [0.52 – 2.99]	0.631
Hispanic	4.44 [2.62 – 7.53]	<0.001	1.40 [0.71 – 2.76]	0.328
**Birthing Parent Race (ref=Multiracial[c])**				
White	0.63 [0.40 – 0.99]	0.049	1.27 [0.62 – 2.62]	0.518
Black	1.73 [1.09 – 2.74]	0.021	1.67 [0.80 – 3.48]	0.173
Combined[b]	0.11 [0.06, 0.20]	<0.001	0.81 [0.35 – 1.94]	0.631
Hispanic	0.49 [0.28 – 0.85]	0.011	1.13 [0.49 – 2.62]	0.775
**Birthing Parent Race (ref=Hispanic)**				
White	1.29 [0.93 – 1.79]	0.127	1.12 [0.71 – 1.79]	0.624
Black	3.51 [2.50 – 4.92]	<0.001	1.48 [0.91 – 2.39]	0.114
Combined[b]	0.23 [0.13 – 0.38]	<0.001	0.71 [0.36 – 1.40]	0.328
Multiracial[c]	2.03 [1.18 – 3.52]	0.011	0.88 [0.38 – 2.05]	0.775

[a]Alternative parameterization of the race and ethnicity group from models shown in Table 2 by changing reference group
[b]Combined group includes selections of American Indian and Alaska Native, Native Hawaiian Asian, and Other race.
[c]Multiracial includes patients self-reporting as two or more race options.
[d]Adjusted model includes covariates of birthing parent age, marital status, insurance, tobacco use, alcohol use, substance use, placental abruption, urine drug testing and neighborhood disadvantage category

© 2023 Schoneich S et al. *JAMA Network Open.*
