# Supplementary material for: Incidence of Newborn Drug Testing and Variations by Birthing Parent Race and Ethnicity Before and After Recreational Cannabis Legalization
Source: JAMA Netw Open. 2023 Mar 8;6(3):e232058. doi: 10.1001/jamanetworkopen.2023.2058 (PMC9996400; doi:10.1001/jamanetworkopen.2023.2058)
Supplement: Supplement 2. — Data Sharing Statement [file jamanetwopen-e232058-s002.pdf]

Data Sharing Statement

Schoneich. Incidence of Newborn Drug Testing and Variations by Birthing Parent Race and Ethnicity Before and After Recreational Cannabis Legalization. *JAMA Netw Open*. Published March 08, 2023. doi:10.1001/jamanetworkopen.2023.2058

Data
Data available: No

Additional Information
Explanation for why data not available: The data include sensitive fields regarding substance use during pregnancy. The authors are happy to connect any interested parties with our institution's data sharing division if there are further questions.
